# Shift-to-Shift Information Transfer: Phenomenological Study of Nurses’ Experiences

**DOI:** 10.2196/81703

**Published:** 2025-11-28

**Authors:** María-Josefa Montoya-Garrido, Claudio-Alberto Rodríguez-Suárez, Noa Mateos-López, Yeray-Tomás Santiago-Díaz, Héctor González-de la Torre

**Affiliations:** 1 Complejo Hospitalario Universitario Insular Materno Infantil, Canary Health Service, Las Palmas de Gran Canaria, Spain; 2 Department of Nursing, Faculty of Health Science, University of Las Palmas de Gran Canaria, Blas Cabrera Felipe, s/n, Las Palmas de Gran Canaria, 35016, Spain, 34 928308880

**Keywords:** communication, nursing, patient handoff, electronic health records, continuity of patient care, patient safety, qualitative research

## Abstract

**Background:**

Handovers represent a critical moment for patient safety, where the effective transfer of information between nurses is essential. In this context, digital documentation systems such as identification, diagnosis, evolution, activities, support (IDEAS) have been implemented to standardize and enhance the quality of clinical handovers.

**Objective:**

This study aims to explore nurses’ perceptions in the hospital setting regarding information transfer during shift changes. Specific objectives included identifying the perceived strengths and weaknesses of the handover process, as well as the difficulties and improvement proposals reported by nurses.

**Methods:**

A qualitative study with a phenomenological approach was conducted. Semistructured interviews were carried out with nurses from the Hospital Universitario Insular de Gran Canaria who had experience using the IDEAS system, between June 2023 and September 2024, until data saturation was reached. After transcribing the interviews, an inductive thematic analysis was performed to identify emerging themes using both descriptive and interpretative approaches. Axial coding through co-occurrence analysis, analytical triangulation, and reflexivity strategies was incorporated to strengthen the credibility and consistency of the findings. Atlas.ti software (version 25.0.1; Scientific Software Development GmbH) was used for the analysis. The study was approved by the local ethics committee (code: 2023-244-1).

**Results:**

From the interviews (n=15), 6 subthemes were identified and grouped into three main themes: (1) nurses (difficulties and improvement proposals in information transfer, strengths and weaknesses in the handover process), (2) patients (electronic health records: Benefit for patients, transfer of patient information), and (3) records (Comments on the form, information management). Participants valued the structured access to clinical information provided by the IDEAS system. However, they reported limitations such as poor data prioritization, editing difficulties, outdated information, and a lack of integration between nursing and medical records. In addition, training deficiencies and variability in system use, particularly among less experienced professionals, were noted. Suggestions for improvement included redesigning the handover form, automating updates, incorporating brief clinical summaries, and providing ongoing training.

**Conclusions:**

While the IDEAS system represents an improvement over previous handover methods, its effectiveness remains constrained by technical, organizational, and cultural barriers. Optimizing the system requires clinically oriented redesigns, alongside training strategies and an institutional culture that promotes shared responsibility for documentation quality. These elements are essential for establishing a safer, more standardized, and patient-centered clinical handover model.

## Introduction

The proper handover of patient care, or the transfer of information regarding a patient’s treatment, is essential to ensure patient safety, reduce unnecessary risks associated with health care delivery [1,2], and maintain continuity of care [3]. The Agency for Healthcare Research and Quality defines this process as a standardized method for transferring information, along with authority and responsibility, during transitions in patient care [1].

In this context, poor communication has been identified as one of the main contributing factors to hospital-related errors. According to Ong and Coiera [4], such errors can occur both during shift changes and intrahospital transfers between different departments. Recommended strategies to improve communication include: formalizing the coordination process prior to transfer without interruptions, planning for discharge in advance, involving the multidisciplinary team actively in the handover, introducing the role of the liaison nurse, increasing available staffing, and implementing a centralized repository to facilitate access to patient information [4].

The standardization of the content and structure of handovers has also been proposed as an effective measure to reduce sentinel events resulting from inaccurate or ineffective communication. These tools involve structured handover models that incorporate checklists to ensure the consistent transmission of essential information, thereby preventing the omission of critical data and contributing to improved clinical safety and patient satisfaction [3]. The scientific literature reports a growing number of such standardized methods [5].

One of the most widely used tools internationally for nurse-to-nurse handovers is the Situation, Background, Assessment, Recommendation (SBAR) protocol, which has been recommended by the Joint Commission International since 2007. However, this model may present limitations in managing patients with complex chronic conditions [5]. Variants of the SBAR protocol have been developed to include additional clinically relevant information. Among them is the Introduction, Situation, Background, Assessment, Recommendation (ISBAR) model [6], which is recommended by the World Health Organization due to its brevity, conciseness, and predictable structure [7,8]. Other variants include the Identify, Situation, Observation, Background, Agreed plan, Read back (iSoBAR) model, developed in Australia, and the Illness severity, Patient summary, Action list, Situation awareness and contingency planning, Synthesis by receiver (I-PASS) model, created in the United States for pediatric settings [5].

The implementation of the ISBAR protocol and its variants has been shown to significantly improve the quality of clinical information transfer, strengthen interprofessional communication, and increase trust among health care professionals. Nurses who use ISBAR report being able to convey patient information in a structured, fast, and efficient manner. Moreover, when professionals have greater access to patient data, they tend to ask more questions and gain a better understanding of the care plan during handover. Thus, the use of this protocol contributes to the development of more effective communication skills in clinical settings [9].

The Identification, Diagnosis, Evolution, Activities, Support (IDEAS) model, in turn, is particularly recommended for information transfer between different health care services or units. This model defines a minimum data set through 5 key components [5].

The use of these structured frameworks for clinical handover has led to improvements in the quality and comprehensiveness of information transfer, in interprofessional collaboration, and in perceived outcomes related to patient safety, quality of care, and health outcomes [10]. However, incomplete handovers and deficiencies in information exchange between health care professionals still persist, which may lead to adverse events for patients [6].

Thus, a properly conducted clinical handover should reduce failures in continuity of care, errors, and patient harm in both hospital and community settings. Nevertheless, in practice, this information transfer is often performed inadequately, omitting critical details and including irrelevant data [8]. Factors such as constant activity, tasks requiring continuity, workload pressure, phone calls, other types of interruptions, and delays in the start of shifts can negatively interfere with the handover process.

Aware of this situation, the Hospital Universitario Insular de Gran Canaria (HUIGC) has adopted the IDEAS model for information transfer during nursing shift changes, based on nurses’ documentation within the electronic health record (EHR). In light of this, the general objective of this study was to explore the perceptions of nurses working in inpatient units at HUIGC regarding the transfer of information during handovers. The specific objectives included identifying the perceived strengths and weaknesses of the handover process, as well as exploring the difficulties encountered and potential areas for improvement as perceived by the nursing staff.

## Methods

### Study Design

A qualitative study was conducted based on the phenomenological paradigm, combining Husserl descriptive perspective [11] with Heidegger interpretive hermeneutics [12].

### Experience or Role of Researchers

The research team was composed of 5 professionals (2 women and 3 men), 3 of whom (MJM-G, NM-L, and YTS-D) were nurses working at HUIGC, with clinical and management experience. The other 2 researchers held PhDs in Nursing and Health Sciences. One of them (CAR-S) worked in the hospital’s research support unit and served as a faculty member at the University of Las Palmas de Gran Canaria (ULPGC), while the second (HGdlT) was also a full professor at ULPGC. Both had previous experience in applying qualitative methodologies. The remaining authors had no prior contact with any of the participating nurses.

### Participants and Sampling

The HUIGC is a tertiary-level university hospital that is part of the Complejo Hospitalario Universitario Insular Materno Infantil, located in the Canary Islands, Spain.

The target population consisted of nurses from various inpatient units who used the IDEAS information transfer model as the method for nursing handover, implemented through the EHR.

Initial participant recruitment was conducted using convenience sampling. Subsequently, to enrich and diversify the range of perspectives, theoretical sampling was used to include new informants who could provide relevant insights into specific aspects of the phenomenon under study [13]. Following the theoretical recommendations of Hennink et al [14], code saturation can typically be reached with 9 participants; however, between 16 and 24 interviews are needed to achieve meaning saturation. Therefore, a minimum sample size of 16 participants was estimated for the study.

Included in the study were nurses working in the inpatient units of HUIGC during the study period, with at least 1 year of clinical experience in that setting. Nurses not directly involved in patient care in inpatient units, such as unit supervisors, area supervisors, or general supervisors, were excluded.

### Data Collection

The interviews were conducted by 2 of the researchers (NM-L and YTS-D) between June 2023 and September 2024. Prior communication was established with participants in order to schedule interviews at times that would not interfere with clinical duties. The interviews were held in quiet, interruption-free spaces within the hospital setting, using offices either within or near the units to avoid unnecessary travel during the participants’ workday.

Relevant sociodemographic variables were collected from key informants, including age, sex, years of professional experience, time spent working in inpatient care, academic qualifications, and typical work shift, in order to contextualize the narratives obtained. In addition, the researchers recorded notes in a field journal. Each interview lasted approximately 30 minutes and was guided by a semi-structured interview script, which is presented in Textbox 1.

Textbox 1. **Semistructured interview guide.**

1. What is your opinion about the current handover model?
2. What advantages would you describe in using the new model compared to the previous way of working?
3. What disadvantages or difficulties have you identified in this new handover model?
4. In your opinion, how would you improve this working model?
5. Have you noticed any lack or absence of information between the EHR data and the new model?
6. Are you familiar with any information transfer models implemented during shift changes at other hospitals where you have worked?
7. Could you describe any errors that have affected patient safety related to the transfer of information, both before and after the implementation of the new model?

### Data Analysis

The interviews were audio-recorded using a mobile phone and subsequently transcribed verbatim after each session. This process was carried out continuously, allowing for a progressive analysis of the data and the identification of relevant excerpts, thus facilitating initial coding and the emergence of analytical categories through an open coding procedure [15].

The analysis followed the classical methodological approach proposed by Glaser and Strauss [16], which involves categorizing information based on the identification of descriptive codes or meaning units (MUs) grouped into emerging analytical categories (subthemes), until theoretical saturation is achieved. Subsequently, a selective coding phase was conducted, in which similar analytical categories were refined and integrated, selecting those most substantiated to identify the core theoretical categories explaining the phenomenon under study. Finally, axial coding was performed through the analysis of category co-occurrences. The analysis of co-occurrences involves identifying and examining the relationships between categories that frequently appear together within the data. This method enables a deeper exploration of how concepts are interconnected in participants’ narratives, uncovering patterns, associations, and emerging themes. By highlighting these interrelations, co-occurrence analysis provides valuable insights into the structure and meaning of the phenomena, enhancing the overall rigor and depth of qualitative analysis [17].

The analysis was conducted using Atlas.ti software (version 25.0.1; Scientific Software Development GmbH). The results were presented through verbatim excerpts and co-occurrence tables, allowing for both descriptive analysis and a deeper interpretation of the data.

### Rigor and Trustworthiness

Once the interviews were transcribed, participants were asked to review and validate the content of the conversations to ensure the accuracy and fidelity of the narratives collected. In addition, data triangulation was applied by comparing the transcriptions with the notes recorded by the researchers in the field journal.

To ensure methodological rigor, the criteria proposed by Lincoln and Guba [18,19] were followed. Credibility was achieved through detailed data collection, which was verified by the informants. Transferability was supported by a thorough description of the setting, participants, context, and methods. Dependability was assessed through external review by an expert (HGdlT) who was not involved in data collection or analysis. Confirmability was established through triangulation between the transcribed data and the researchers’ field notes, as well as through reflexivity regarding the researchers’ own potential biases.

Furthermore, the study adhered to the guidelines of the Consolidated Criteria for Reporting Qualitative Research (COREQ) [20], which establish standards for quality and transparency in qualitative studies.

### Ethical Considerations

All ethical principles concerning confidentiality, anonymity, and the exclusive use of data for research purposes were strictly upheld throughout the study. The collection, processing, and storage of personal data complied with current legislation. Moreover, the fundamental ethical principles outlined in the Nuremberg Code, the Declaration of Helsinki, and the Belmont Report were observed, ensuring informed consent, the right to autonomous decision-making, and the protection of participants at all times. The study was approved by the Research Ethics Committee of the Hospital Universitario Doctor Negrín de Gran Canaria, Las Palmas (code 2023-244-1).

## Results

### Participant Characteristics

Of the 16 informants, 1 withdrew their participation after the interview was conducted; therefore, only 15 participants were included in the analysis. Their sociodemographic characteristics are presented in Table 1.

**Table 1.** Sociodemographic characteristics of participants.

Participants (P)	Sex	Age (years)	Years of experience	Years in unit	Academic degree	Employment type	Work shift
P1	Female	43	20	15	Master’s	Permanent	Day shift (8 h)
P2	Male	35	9	3	Grade	Interim	Day shift (8 h)
P3	Female	35	14	4	Grade	Interim	Rotating shift (12 h)
P4	Male	39	11	11	Grade	Interim	Rotating shift (12 h)
P5	Female	25	3	3	Grade	Temporary	Rotating shift (12 h)
P6	Female	33	12	6	Grade	Interim	Rotating shift (12 h)
P7	Female	41	20	20	Master’s	Interim	Rotating shift (12 h)
P8	Female	42	21	10	Master’s	Permanent	Rotating shift (12 h)
P9	Female	42	21	20	Diploma	Permanent	Morning shift (8 h)
P10	Female	37	9	9	Grade	Interim	Rotating shift (12 h)
P11	Female	46	25	23	Diploma	Permanent	Rotating shift (12 h)
P12	Female	26	4	2	Grade	Temporary	Rotating shift (12 h)
P13	Female	35	5	3	Master’s	Temporary	Rotating shift (12 h)
P14	Female	43	10	8	Master’s	Interim	Rotating shift (12 h)
P15	Female	33	11	10	Grade	Interim	Rotating shift (12 h)

### Themes

The content analysis included a total of 173 MUs, distributed across 6 subthemes, which were grouped into three overarching themes: (1) nurses (difficulties and improvement proposals in information transfer, n=27 MUs; strengths and weaknesses in the handover process, n=19 MUs); (2) patients (EHR: benefit for patients, n=22 MUs; transfer of patient information, n=21 MUs); and (3) records (comments on the form, n=30 MUs; information management, n=54 MUs). The main themes and subthemes are presented in Table 2, and the full coding tree is available in Multimedia Appendix 1.

**Table 2.** Themes and subthemes.

Themes	Subthemes
Nurses	Difficulties and improvement proposals in information transfer Strengths and weaknesses in the handover process
Patients	Electronic health records: benefit for patients Transfer of patient information
Records	Comments on the form Information management

#### Theme 1: Nurses

##### Difficulties and Improvement Proposals in Information Transfer

Nurses’ perceptions revealed a mismatch between the design of the handover form and the actual needs of those using it daily.

Although the system provided a structured framework, many professionals perceived it as impractical and overloaded with data of limited immediate value, which made interpretation difficult, especially in high workload contexts.

Among the main issues identified were the presence of excessive or irrelevant information and unclear visual organization, both of which hindered a fast and effective handover:

I want it to be as simple as possible and something visual, quick… I want to know the patient’s diagnosis, how their day has gone, the most important things.

[P7]

I’m not interested in you telling me there’s a blood test and that there’s a red tube, green tube…

[P7]

A recurring complaint was the persistence of outdated clinical records, such as removed catheters or drains, which continued to appear in the form. This lack of updating generated confusion and increased the risk of clinical errors:

You remove the drain… but that handover information never gets deleted…

[P7]

In addition, the care search system was described as inefficient and unintuitive, making it difficult to locate or update care entries. The inclusion of irrelevant elements for certain units was also criticized, as it cluttered the system without adding value:

The search tool is super crappy… you end up typing it yourself, otherwise it takes forever…

[P7]

I get newborn care options… I work with adults, I don’t need to bathe a newborn.

[P7]

Another significant limitation identified was the lack of proper nursing assessments, which compromised the quality of the handover and highlighted the need for greater professional involvement in recordkeeping:

Nurses don’t do the assessment properly… they don’t update the care plans…

[P4]

Improvement proposals were organized around three main areas: reviewing content, automating the updating of care records, and reorganizing the system’s visual and functional layout. Regarding the first area, participants offered several suggestions to optimize the usefulness and relevance of the recorded content:

It should be divided into sections, and the information we need should be highlighted: vital signs, oxygen, care, diagnosis… background in a summarized way.

[P7]

For me, it should be more concise, I’m a very structured and simple person…

[P4]

Participants also suggested highlighting priority care and presenting information in a more structured and concise format to allow for quicker reading and better decision-making during handovers:

Maybe they could prioritize the important background items, because the truth is, they often include a whole lot of history… hypertensive, diabetic, and I don’t know, a prior stroke or something more relevant.

[P2]

I would highlight the care section… it would help me, especially if I’m new to that place.

[P10]

Redundant or irrelevant information was also targeted for removal, with an emphasis on prioritizing the most critical elements for immediate care:

First things first: vital signs, care, patient diagnosis, summarized background…

[P7]

Another key issue was the need for the system to automatically remove suspended interventions or care entries from the record, to prevent confusion caused by outdated information:

When you remove the IV line, it shouldn’t still appear… same with the urinary catheter…

[P7]

They shouldn’t appear anymore once you’ve discontinued them on the form…

[P9]

Despite the identified shortcomings, several participants acknowledged that the current system represented progress compared to traditional paper-based handovers, as it centralized information and allowed for updated summaries. Among the specific suggestions, participants recommended making certain key data more visible for daily management, such as the patient’s medical record number or contact phone:

It wouldn’t hurt to have the patient’s phone number appear… the medical record number in a larger font…

[P8]

Finally, participants proposed redesigning the medical orders section to limit its content to active instructions, removing obsolete or duplicate entries, thereby facilitating a quicker and more accurate interpretation of the patient’s clinical status:

The medical orders section is useless… you don’t see the referrals or the canceled blood tests…

[P7]

##### Strengths and Weaknesses in the Handover Process

The implementation of the digital system for handover represented progress in terms of speed, standardization, and access to clinical information, helping to prevent duplicated tasks and improve continuity of care. However, digitalization alone did not guarantee a safe and efficient handover if human and organizational factors were not considered. Its effectiveness depended on the staff’s commitment to keeping the information updated and aligned with the patient’s actual condition, particularly in settings with high turnover or the onboarding of new professionals. To fully capitalize on the benefits of technology, it was necessary to redesign the system’s format, enhance its interoperability with other clinical tools, and promote a shared culture of responsibility regarding the quality of information.

Although nurses appreciated having a structured document to guide the transmission of information, they warned that overreliance on written support could reduce the rigor of verbal communication:

…overall, the information is pretty good because it serves as a guide.

[P10]

Not so much the model itself, but… at the nursing level, I think people get too comfortable sometimes, knowing that everything’s written down…

[P10]

In terms of strengths, one of the most valued improvements was the automation of data entry, which reduced the time required to prepare the handover report:

It saves a lot of time… before you had to keep changing, modifying, and it was extra work…

[P8]

Unified access to information allowed for the generation of a centralized document with updated clinical data, facilitating review and printing at the beginning of the shift. This prevented duplicate entries and enabled quicker reading:

With this handover, you already have everything uploaded… you update the care plans and print it directly…

(P7]

Content standardization also contributed to greater uniformity in format, reducing the risk of omissions or errors due to subjective interpretations:

It has made things easier… everything is uploaded automatically now…

[P8]

Among the main weaknesses, participants pointed out the persistence of outdated information, such as devices or treatments that had already been discontinued, which led to confusion and potential clinical errors:

We remove a basic catheter and it still shows up… old information.

[P9]

In the handover it still shows that they have an IV line from July 2nd…

[P7]

Another issue was the overload of poorly prioritized content, making it difficult to quickly identify what was essential for immediate care:

I just want to know the patient’s name, diagnosis, and what I need to do for them…

[P7]

Honestly, personal history… more concise, more visual.

[P4]

The system also offered little flexibility to edit records, which forced nurses to delete and re-enter information:

You should be able to edit the care entries… instead of deleting the whole thing.

[P7]

Another relevant aspect was the reliance on individual professional judgment to keep records up to date, which created variability in the quality of handovers:

You’re not going to write that the patient needs their nails cut… if they’re being discharged tomorrow.

[P7]

People get too comfortable… since it’s already written, they think it’ll be read and don’t put as much effort into explaining the shift.

[P10]

Finally, participants identified shortcomings in the visibility of critical information, such as prescribed medications or active medical orders, which complicated decision-making during handovers:

The medication doesn’t appear… even insulin prescriptions, you have to go looking for them.

[P10]

### Theme 2: Patients

#### EHRs: Benefit for Patients

Overall perceptions of the IDEAS system were predominantly positive regarding its contributions to patient care. Nurses appreciated the quick and centralized access to key clinical data, which facilitated decision-making and contributed to more agile and safer care. The ability to consult patient history, treatments, and vascular access instantly was highlighted as a significant advantage:

…you have the vascular access there and all the basic and necessary information you need to talk about the patient.

[P4]

The system was also perceived as an improvement over previous models, both in terms of time-saving and the organization of the handover:

…you don’t have to keep duplicating information or re-entering everything again…

[P7]

The existence of a common and structured support system helped ensure continuity of care and minimized the risk of information loss between shifts:

…you see the diagnoses more concisely, the care measures more highlighted, so you kind of know how to guide yourself with the patient.

[P2]

In addition, IDEAS allowed for a quick understanding of a patient’s clinical status, even when the nurse had not previously cared for them:

…a mini patient progress summary would really help when you don’t know the patient.

[P8]

Having essential information such as allergies, critical history, or active care plans readily available provided a sense of security, especially in situations that required quick decision-making:

…you have that handover in front of you with your care plans, allergies… in the end, that’s something important you need to have there…

[P10]

Among the main limitations, participants identified information overload and dispersion, particularly in the patient history and care sections, where excessive or irrelevant data were often included:

…you might get half a page of history… when the basics would just be hypertension, diabetes…

[P4]

They also questioned the frequent use of “copy and paste” without individualized assessment:

…all the care entries are just dumped in without evaluating the patient… you have to delete what isn’t useful.

[P7]

Despite these shortcomings, the system was acknowledged as an improvement in terms of efficiency, organization, and reliability:

…I’ve rated it so positively… because it’s organized and makes it easier to give the handover.

[P10]

Nevertheless, participants stressed the need to simplify the format, make it more visual, and better adapt it to the characteristics of the patient and the workflow of each unit:

…so many words all over the place that you wonder: what am I actually supposed to focus on?

[P7]

#### Transfer of Patient Information

The transfer of patient information was perceived as a key aspect of care quality, and the IDEAS system introduced notable improvements compared to previous models. One of the most valued features was the ability to quickly identify priority care tasks according to the patient’s clinical condition:

Care priorities, I mean, the first thing they tell you during the handover, how the patient is doing overall, the care areas where you have to focus more…

[P2]

Participants highlighted the need for a more visual, hierarchical, and concise format that would allow quick access to essential information without having to read long texts:

…so that the handover makes the information easier to access.

[P2]

The integration of the system with the EHR was also appreciated, as it ensured traceability, legal consistency, and data reliability:

This model comes directly from the Drago program, which is supposed to be legal, and when you enter all the correct information, everything that’s reflected in the patient record shows up in the handover.

[P4]

This structure also helped reduce errors associated with overreliance on verbal communication:

Sometimes the information wasn’t accurate… and what my colleague told me often had nothing to do with reality, important data were omitted.

[P5]

Nevertheless, several limitations were noted, such as the difficulty of retrieving relevant information when managing multiple patients, particularly when data were poorly organized:

It’s not functional at all, not practical… you waste a lot of time searching, because of course… you’ve got 14 patients…

[P7]

Another weakness was the lack of integration between different professionals, especially between doctors and nurses, which led to gaps in information essential for care planning:

Sometimes the information doesn’t come through completely… and I would highlight the nurse’s shift note, the latest blood pressure readings, the glucose levels…

[P10]

In addition, there were cases where information failed to appear in the handover report due to incomplete documentation in the relevant forms:

If it’s not filled out in the forms, then it won’t show up in the printed handover.

[P2]

Some nurses expressed a preference for more functional formats that allowed them to focus on the most essential aspects of the patient:

When I take over the shift, I write down on a sheet what really matters to me about the patient (diagnosis, care).

[P4]

Others noted that when the system was used properly, it reduced the time spent drafting the handover report and allowed more focus on direct patient care:

They don’t put as much effort into explaining the handover… since everything is written down, it’s like: there it is, you’ll read it.

[P10]

Overall, the system was positively evaluated. Improvements were acknowledged in workflow organization, information accessibility, and the quality of the handover. However, participants emphasized the need for enhancements in data visualization, content simplification, and adaptation to the specific needs of nursing care and interdisciplinary collaboration:

…I would highlight a few more things related to nursing… I think it would be helpful and would make our work easier, especially when starting the daily care routine.

[P10]

I like the model we’re using, I see it as an advantage.

[P10]

### Theme 3: Records

#### Comments on the Form

The IDEAS form was valued for its ability to integrate a large amount of clinical information, offering a comprehensive and detailed view of the patient’s condition. However, this wealth of data did not always prove functional in daily practice due to the lack of content prioritization and a poorly structured visual layout, which hindered the quick identification of key elements for immediate care. Technical limitations were noted, including difficulties in editing errors, the rigidity of the form’s design, and limited usability, all of which discouraged efficient use:

If I make a mistake in one word, I have to delete the whole thing to… do it again, and the forms look terrible…

[P7]

If we all updated everything, the shift report itself would be much more complete and efficient.

[P7]

Despite these difficulties, the system was perceived as an improvement over verbal handovers or handwritten formats. Its structure and level of detail helped reduce errors due to omission and improved continuity of care:

The information is quite good because it serves as a guide for us.

[P10]

To enhance the tool, participants agreed on the need for a more agile model, with better content prioritization, increased interoperability among professionals, and less unnecessary informational load. Suggested improvements included enabling editing without having to delete the entire record, summarizing nonpriority information, and synchronizing forms across clinical roles:

I should be able to summarize it and keep it that way, not depend solely on what the doctor has written…

[P7]

If you fill out the forms properly with the drains, the IV lines… all you need to write is how the patient is doing.

[P7]

#### Information Management

One of the most frequently reported weaknesses was the lack of record updating. In many cases, discontinued interventions or care measures remained visible in the system, leading to confusion and increasing the risk of clinical errors:

We remove a drain and it still shows up…

[P9]

Not everyone reviews them… not everyone updates them…

[P8]

Limited interoperability between different areas of the system and among professional roles was also noted. The lack of connection between what physicians recorded and what was accessible to nurses or nursing assistants created fragmentation:

It would be better if the care plans created by physicians were transferred directly to the assistants’ section.

[P10]

The excess of redundant information, without a clear prioritization structure, complicated access to relevant data and increased the cognitive load on staff:

In the same box, it shows the IV line, the catheter, then the diet—everything is a bit mixed up…

[P7]

I would structure it so that the most necessary information stands out: vital signs… care, diagnosis…

[P7]

Technical difficulties were also cited regarding the modification of care plans, which required deleting the entire entry instead of editing, hindering the correction of errors:

Every time there’s an admission, you have to enter all the information… you have to close Word and can’t type…

[P7]

Another reported limitation was the lack of synchronization between medical diagnoses and nursing functional patterns, affecting the consistency of the clinical record:

The medical diagnosis changes… and the nursing one hasn’t been updated…

[P8]

Despite these issues, professionals positively valued the IDEAS system as an improvement over previous handover models, emphasizing its contribution to preventing information loss and facilitating continuity of care:

The current format for handover is useful, since it summarizes many aspects of the patient.

[P4]

### Co-Occurrences

The co-occurrence analysis revealed the highest number of relationships between the subthemes information management and comments on the form (n=84), followed by information management and strengths and weaknesses in the handover process (n=82). The remaining co-occurrences are presented in Table 3.

**Table 3.** Co-occurrence analysis between subthemes.

Subthemes	Comments on the form (n)	Difficulties and improvement proposals in information transfer (n)	Strengths and weaknesses in the handover process (n)	Information management (n)	Electronic health record: Benefit for patients (n)	Transfer of patient information (n)
Comments on the form	0	57	71	84	64	59
Difficulties and improvement proposals in information transfer	57	0	66	67	46	39
Strengths and weaknesses in the handover process	71	66	0	82	56	52
Information management	84	67	82	0	58	58
Electronic health record: Benefit for patients	64	46	56	58	0	56
Transfer of patient information	59	39	52	58	56	0

## Discussion

### Principal Findings

The transfer of clinical information between nursing professionals during shift changes remains a critical process to ensure patient safety and continuity of care [5]. In this study, tensions were identified between the perceived benefits of the IDEAS system and its functional and organizational limitations. While this digital tool is recognized for centralizing access to key information, participants noted that structural and functional barriers persist, hindering clear, accurate, and useful communication. These findings align with previous studies that have highlighted how fragmented records, lack of standardization, excessive irrelevant or outdated information, and system rigidity compromise the quality of handovers and increase the risk of clinical errors [4,21,22].

From an organizational perspective, structured communication models such as SBAR, ISBAR, or IDEAS have proven effective by providing hierarchical and concise frameworks for transmitting essential data [9,23]. In the present study, nurses acknowledged that IDEAS has facilitated the automation of documentation and the centralization of information, thereby improving the efficiency of the process [10]. Unlike SBAR, ISBAR, or I-PASS, which primarily focus on verbal handovers, IDEAS emphasizes integration with the EHR, introducing unique challenges related to information accuracy, workflow adaptation, and usability. This distinction helps contextualize the specific benefits and limitations identified in this study, including the persistence of outdated data, difficulties in editing information, and a lack of flexibility—factors consistent with barriers previously reported in the literature, such as limited interoperability between records and the influence of organizational culture on effective system use [3,22].

Regarding the content of handovers, participants emphasized that IDEAS has improved accessibility to essential clinical data, such as diagnoses, vital signs, active care plans, and medication. However, difficulties were noted related to the overload of irrelevant or outdated information, which compromises the clarity of the clinical message and hinders decision-making. Nurses expressed the need for a simpler, visually clear, and sectioned format, focused on priority aspects such as diagnosis, vital signs, and active care. This need aligns with previous research showing that content simplification supports faster and safer decision-making [8,21], particularly through the use of standardized summaries that prioritize critical information [24,25].

Particularly, the usefulness of information emerged as a crosscutting MU that contributed relevant nuances to all identified subthemes and themes. In this regard, nurses appreciated having access to data on patients’ medical history, vascular access, and recent clinical evolution, as these elements help build a comprehensive understanding of the patient’s condition and facilitate care planning. This perception aligns with other studies emphasizing how concise and relevant information enhances clinical efficiency [24,26]. However, participants proposed incorporating brief clinical summaries to synthesize the patient’s evolution, especially valuable when caring for unfamiliar patients, an idea that has also been validated in models such as I-PASS and iSoBAR [7,27].

A transversal criticism of the handover content concerned the inability to prioritize or rank clinical problems within the IDEAS system. Participants emphasized the need for a structured, visual, and concise model that would facilitate the rapid identification of the most relevant clinical data, tailored to the specific characteristics of each unit and the level of care burden. This proposal aligns with the demonstrated effectiveness of frameworks such as SBAR and ISBAR, which allow for the hierarchical organization of information centered on the patient’s clinical status [9,23]. In addition, nurses highlighted the need to incorporate a more holistic view of care, including emotional and social dimensions of the patient—an aspect still insufficiently reflected in digital records, yet considered essential for comprehensive care [3,27].

### Practical and Research Implications

Regarding the training received, a common concern that emerged was the absence of formal instruction on how to conduct clinical handovers. This gap results in considerable variability in clinical practice and is recognized as a risk factor for patient safety, particularly among professionals with less clinical experience [28,29]. Participants highlighted the value of being accompanied by a supervising professional during their early clinical experiences, underscoring the importance of guided mentorship in the development of both communicative and clinical skills, as previously emphasized by Martínez-Fernández et al [30] and Tacchini-Jacquier et al [31].

Complementarily, the incorporation of innovative educational strategies, such as virtual reality and role-playing, has been proposed for teaching structured models like ISBAR. Recent literature supports this approach, showing that such methodologies can enhance perceived competence, knowledge retention, and the confidence of novice professionals [29,32]. Moreover, these tools may help reduce variability in the execution of clinical handovers, even in settings where digital systems have already been implemented.

From the patient’s perspective, the quality of clinical handover directly impacts their care experience. Nurses expressed appreciation for the ability to quickly access key information, such as medical history, current care plans, or vascular access, in line with studies that associate the use of structured systems with increased perceptions of safety and quality of care [6,26]. However, the lack of integration between medical and nursing records, particularly regarding nonpharmacological care, remains a critical limitation. This disconnect hinders a holistic understanding of the patient and compromises continuity of care [3,4].

From a health care management perspective, the implementation of tools such as SBAR has not only contributed to the reduction of incidents related to communication failures but also improved perceptions of safety and interprofessional collaboration [27,30]. However, for their implementation to be effective, these tools must be supported by complementary strategies, including ongoing training, regular audits, feedback mechanisms, and an institutional culture that recognizes clinical handover as a core component of patient safety [31,32].

Overall, although the IDEAS system represents a significant improvement over previous paper-based methods, structural, content-related, and organizational barriers persist that limit its potential impact. Participants proposed several practical improvements to enhance the system, which can be grouped into three categories:

1. Technical interventions, including automated updates to ensure information accuracy and reduce errors.
2. Organizational interventions, such as standardizing workflows and integrating information across different units, to improve usability and continuity of care.
3. Training interventions, encompassing structured guidance during early clinical experiences, mentorship, and the incorporation of innovative educational strategies like virtual reality and role-playing to strengthen competence and confidence in clinical handovers.

These interventions, together with an organizational culture focused on patient safety, emerge as key elements to achieve an effective, safe, and care-centered handover process.

### Strengths and Limitations

One of the main strengths of this study lies in its phenomenological approach, which enabled an in-depth exploration of nurses’ subjective experiences, revealing complex and contextual meanings that often remain hidden in quantitative research. In our analysis, Husserl descriptive phenomenology guided the initial coding of nurses’ lived experiences, while Heidegger hermeneutics informed the interpretative stage, enabling us to situate these experiences within broader contextual and existential dimensions of nursing practice. The use of semistructured interviews facilitated openness and reflection among participants, enriching the quality of the data collected. Furthermore, the inclusion of diverse professional profiles (in terms of experience, seniority, and clinical units) provided a broader and more nuanced perspective on the phenomenon under study. The application of analytical triangulation strategies, along with peer review and critical reflection by the research team, contributed to enhancing the credibility and interpretative coherence of the analysis.

However, this study presents some limitations inherent to its phenomenological design. By focusing on the subjective experiences of a small group of nurses within a specific clinical context, the findings are not generalizable to other units, institutions, or organizational settings. Participants were drawn from different general hospitalization units where the IDEAS system was implemented; however, no staff from specialized units (eg, intensive care unit) were included, and specific unit information was omitted to maintain participant anonymity. Therefore, future research should include specialized units and other institutions to enhance the transferability of these findings. The purposive sampling strategy allowed for a rich and diverse description of the phenomenon, but it also entails a potential selection bias, as those who agreed to participate may have had a particular interest in or perspective on clinical handover.

Similarly, the interpretation of the data was influenced by the interaction between the researchers and the participants. While this is inherent to the phenomenological approach, it must be acknowledged as part of the research team’s reflexive positioning. Although methodological strategies aimed at enhancing credibility and triangulation of the analysis were applied, the inherent subjectivity of the qualitative approach may influence the construction of categories and the interpretation of meanings. These limitations should be considered when assessing the scope, transferability, and applicability of the findings.

### Conclusions

The IDEAS system has represented a significant step forward in structuring clinical handovers, improving access to and organization of information during shift changes. However, technical and organizational limitations remain that hinder its effectiveness, such as the lack of data prioritization, the presence of outdated information, and limited integration between medical and nursing records.

The experiences collected highlight a gap between the current design of the system and the real needs of nurses. To optimize its usefulness, the form must be redesigned toward a more visual, editable format focused on clinically relevant content. Proposed improvements include the automatic update of inactive care plans, the incorporation of brief clinical summaries, and increased visibility of key data.

Moreover, the system’s effectiveness depends not only on technical enhancements but also on institutional commitment to training, a culture of safety, and shared responsibility in the use of clinical records. Integrating these elements will foster a safer, more efficient, and patient-centered handover process.

## Supplementary material

10.2196/81703 Multimedia Appendix 1 Coding tree.

## Abbreviations

COREQ: Consolidated Criteria for Reporting Qualitative Research
EHR: electronic health record
HUIGC: Hospital Universitario Insular de Gran Canaria
I-PASS: Illness severity, Patient summary, Action list, Situation awareness and contingency planning, Synthesis by receiver
IDEAS: Identification, Diagnosis, Evolution, Activities, Support
ISBAR: Introduction, Situation, Background, Assessment, Recommendation
iSoBAR: Identify, Situation, Observation, Background, Agreed plan, Read back
MU: meaning unit
SBAR: Situation, Background, Assessment, Recommendation

## References

[1] McCarthy S, Motala A, Lawson E, Shekelle PG (2025). Use of structured handoff protocols for within-hospital unit transitions: a systematic review from making healthcare safer IV. BMJ Qual Saf.

[2] Müller M, Jürgens J, Redaèlli M, Klingberg K, Hautz WE, Stock S (2018). Impact of the communication and patient hand-off tool SBAR on patient safety: a systematic review. BMJ Open.

[3] Ghosh S, Ramamoorthy L, Pottakat B (2021). Impact of structured clinical handover protocol on communication and patient satisfaction. J Patient Exp.

[4] Ong MS, Coiera E (2011). A systematic review of failures in handoff communication during intrahospital transfers. Jt Comm J Qual Patient Saf.

[5] Alcalá Minagorre PJ, Domingo Garau A, Salmerón Fernández MJ (2023). Safe handoff practices and improvement of communication in different paediatric settings. An Pediatr (Engl Ed).

[6] Lazzari C (2024). Implementing the verbal and electronic handover in general and psychiatric nursing using the introduction, situation, background, assessment, and recommendation framework: a systematic review. Iran J Nurs Midwifery Res.

[7] Randmaa M, Mårtensson G, Leo Swenne C, Engström M (2014). SBAR improves communication and safety climate and decreases incident reports due to communication errors in an anaesthetic clinic: a prospective intervention study. BMJ Open.

[8] Burgess A, van Diggele C, Roberts C, Mellis C (2020). Teaching clinical handover with ISBAR. BMC Med Educ.

[9] Pun J (2021). Factors associated with nurses’ perceptions, their communication skills and the quality of clinical handover in the Hong Kong context. BMC Nurs.

[10] Moyo P, Anderson J, Francis K, Biles J (2024). Exploring the experiences and perceptions of the utilisation of structured clinical handover frameworks by nurses working in acute care settings: a scoping review. J Clin Nurs.

[11] Dowling M (2007). From Husserl to van Manen. A review of different phenomenological approaches. Int J Nurs Stud.

[12] Mendieta-Izquierdo G, Ramírez-Rodríguez JC, Fuerte JA (2015). La fenomenología desde la perspectiva hermenéutica de Heidegger: una propuesta metodológica para la salud pública [Article in Spanish]. Rev Fac Nac Salud Pública.

[13] Moser A, Korstjens I (2018). Series: practical guidance to qualitative research. Part 3: sampling, data collection and analysis. Eur J Gen Pract.

[14] Hennink MM, Kaiser BN, Marconi VC (2017). Code saturation versus meaning saturation: how many interviews are enough?. Qual Health Res.

[15] Bover A (2013). Herramientas de reflexividad y posicionalidad para promover la coherencia teórico-metodológica al inicio de una investigación cualitativa [Article in Spanish]. Enfermería Clínica.

[16] del Moral G, Suárez-Relinque C (2020). La categorización familiar como técnica de apoyo al proceso de análisis que sigue la teoría fundamentada [Article in Spanish]. Gac Sanit.

[17] Scharp KM (2021). Thematic co-occurrence analysis: advancing a theory and qualitative method to illuminate ambivalent experiences. J Commun.

[18] Lincoln YS, Guba EG (1986). But is it rigorous? Trustworthiness and authenticity in naturalistic evaluation. New Dir Program Eval.

[19] Nowell LS, Norris JM, White D, Moules NJ (2017). Thematic analysis: striving to meet the trustworthiness criteria. Int J Qual Methods.

[20] Tong A, Sainsbury P, Craig J (2007). Consolidated criteria for reporting qualitative research (COREQ): a 32-item checklist for interviews and focus groups. Int J Qual Health Care.

[21] Flemming D, Hübner U (2013). How to improve change of shift handovers and collaborative grounding and what role does the electronic patient record system play? Results of a systematic literature review. Int J Med Inform.

[22] Kruse CS, Marquez G, Nelson D, Palomares O (2018). The use of health information exchange to augment patient handoff in long-term care: a systematic review. Appl Clin Inform.

[23] Reime MH, Tangvik LS, Kinn-Mikalsen MA, Johnsgaard T (2024). Intrahospital handovers before and after the implementation of ISBAR communication: a quality improvement study on ICU nurses’ handovers to general medical ward nurses. Nurs Rep.

[24] Sun YK, Shih WC, Cheng KH (2018). An electronic handover system to improve information transfer for surgical patients. Comput Inform Nurs.

[25] Abraham J, King CR, Pedamallu L, Light M, Henrichs B (2024). Effect of standardized EHR-integrated handoff report on intraoperative communication outcomes. J Am Med Inform Assoc.

[26] Michaud-Hamilton N, Frisch N, Roudsari A (2015). Nursing handover using an electronic application for community nurses. Stud Health Technol Inform.

[27] Kullberg A, Sharp L, Dahl O, Brandberg Y, Bergenmar M (2018). Nurse perceptions of person-centered handovers in the oncological inpatient setting: a qualitative study. Int J Nurs Stud.

[28] Gheisari F, Farzi S, Tarrahi MJ (2025). The effect of an ISBAR-based clinical supervision model during handover on clinical decision-making and self-efficacy of nursing internship students: a quasi-experimental study. BMC Med Educ.

[29] Andreasen EM, Høigaard R, Berg H, Steinsbekk A, Haraldstad K (2022). Usability evaluation of the preoperative ISBAR (identification, situation, background, assessment, and recommendation) desktop virtual reality application: qualitative observational study. JMIR Hum Factors.

[30] Martínez-Fernández MC, Castiñeiras-Martín S, Liébana-Presa C, Fernández-Martínez E, Gomes L, Marques-Sanchez P (2022). SBAR method for improving well-being in the internal medicine unit: quasi-experimental research. Int J Environ Res Public Health.

[31] Tacchini-Jacquier N, Hertzog H, Ambord K, Urben P, Turini P, Verloo H (2020). An evidence-based, nursing handover standard for a multisite public hospital in Switzerland: web-based, modified Delphi study. JMIR Nurs.

[32] Brown-Deveaux D, Kaplan S, Gabbe L, Mansfield L (2022). Transformational leadership meets innovative strategy: how nurse leaders and clinical nurses redesigned bedside handover to improve nursing practice. Nurse Lead.

