# Supplementary material for: Shift-to-Shift Information Transfer: Phenomenological Study of Nurses’ Experiences
Source: JMIR Nurs. 2025 Nov 28;8:e81703. doi: 10.2196/81703 (PMC12674989; doi:10.2196/81703)
Supplement: Multimedia Appendix 1 [file nursing-v8-e81703-s001.docx]

**Table S1.**

| Themes (T) | Subthemes | Units of Meaning |
| --- | --- | --- |
| T1: Nurses | Difficulties and improvement proposals in information transfer | Challenges  Clarity  Confusion  Difficulties  Editing  Electronic Health Records  Enhancement  Errors  Improvement Suggestions  Improvements  Information Organization  Information Transfer  Labeling  Lack of Organization  Lack of structure  Organization  Outdated information  Patient Care  Patient Safety  Personal History  Personalization  Poor organization  Poor structure  Process Improvement  Proposal  Safety  Training |
|  | Strengths and weaknesses in handover process | Clarity  Confusion  Electronic Health Records  Errors  Identification  Improvement suggestions  Improvements  Information organization  Lack of Organization  Negative Impact  Outdated information  Patient care  Patient care improvement  Patient Safety  Potential Impact  Strengths  Strengths and weaknesses  Suggestions for improvement  Weaknesses |
| T2: Patients | Electronic health records: Benefit for patient | Benefit to Patient Care  Clarity of care  Editable patient care information  Electronic health record system  Electronic health record Access  Electronic health record strengths  Electronic health record weaknesses  Enhanced patient care and safety  Enhancement for patient care  Formularies  Impact on Care  Improved patient information transfer  Medical Devices  Nursing Care  Patient Care Benefits  Patient Care Improvement  Patient Care Information Transfer  Patient Safety Enhancement  Perception of nurses  Summary of clinical history  Urinary Catheters  Weaknesses in Electronic medical record |
|  | Transfer of patient information | Accuracy  Areas for Improvement  Clarity  Confusion  Deficiencies  Errors  Improvement suggestions  Information Transfer  Lack of information  Lack of structure  Need for improvement  Outdated information  Patient Safety  Perception  Poor structure  Positive opinion  Positive Perception  Risks  Suggestions for improvement  Unclear  Usefulness |
| T3: Records | Comments on the form | Challenging aspects  Confusing  Difficult to understand  Editing Difficulties  Editing Weakness  Form Structure  Forms  Helpful Aspects  Identified difficulties  Impactful Weaknesses  Lack of Structure  Mixed opinion  Mixed Perception  Not easy to understand  Not Useful  Organized system  Personal Antecedents  Poor Form Design  Poor Organization  Poor Structure  Potential Errors  Potential Strengths  Removed Drains  Section separation proposal  Separation of Sections  Shift changes  Shift model  Signaling  Strengths and Weaknesses  Useful Tool |
|  | Information management | Accurate Updating  Alert System  Care Information Editing  Changes Needed  Clarity of shift change process  Data duplication  Direct Editing  Duplicated Information  Editability  Editing Ability  Editing Care Information  Editing difficulties in care information  Editing Errors  Editing Feature  Editing Proposals  Enhanced Information Transfer Process  Enhancement proposals  Enhancing utility  Impact on Shift Change  Improvement Opportunities  Improvement Proposals  Improvements Benefit  Improvements Needed  Information Editing  Information Precision  Information Presentation  Information Separation  Information transfer  Information Transfer Challenges  Information Transfer Enhancement  Information Transfer Improvement  Information Transfer Process  Information Updating  Lack of Updating  Outdated Information  Outdated information retention  Outdated Information.  Potential Changes  Potential Improvement  Process Enhancement  Process Improvement Proposals  Proper Updating  Proposals for improvement  Proposed Enhancements  Proposed Improvements  Relevant Information  Shift Change Process  Signalization  Standardized model  Standardized Shift Change  Standardized Shift Change Model  Suggestions for Change  System updates  Updated Information |
